# Adverse Childhood Experiences, Positive Childhood Experiences, and Digital Media Use among Children and Adolescents in the United States

**DOI:** 10.1007/s40653-025-00785-z

**Published:** 2025-10-21

**Authors:** Elizabeth Crouch, Cheri Shapiro, Emma Boswell, Monique Brown, Kevin J. Bennett

**Affiliations:** 1 School of Medicine, Department of Family and Preventive Medicine, University of South Carolina, Columbia, USA; 2 Department of Health Services Policy & Management, University of South Carolina, Columbia, USA; 3 Institute for Families in Society, College of Social Work, University of South Carolina, Columbia, USA; 4 University of South Carolina Rural Health Research Center, University of South Carolina, Columbia, USA; 5 South Carolina SmartState Center for Healthcare Quality, Arnold School of Public Health, University of South Carolina, Columbia, USA

**Keywords:** Digital media, Adverse childhood experiences, Positive childhood experiences; resilience

## Abstract

Compensatory internet use has been demonstrated to be a potential coping mechanism for stressors, which may lead to heavy digital media use. Little is known about what family-based factors may contribute to heavy digital media use among children and adolescents. This study used cross-sectional data from the 2021–2022 National Survey of Children’s Health to examine the prevalence of adverse and positive childhood experiences (ACEs and PCEs) and how these rates may vary by light, moderate, and heavy digital media use among children and adolescents in a nationally representative sample in the U.S (*n* = 52,256). Bivariate analyses were used to estimate prevalences and multivariable regression models were used to examine the relationship between ACEs, PCEs and heavy digital media use. Children who engaged in heavy digital media use were more likely to report each type of ACE than children who engaged in light digital media use (*p* < 0.0001). Alternatively, children who engaged in heavy digital media use were less likely to report each type of PCE than children who engaged in light digital media use (*p* < 0.0001). Children who had experienced four or more ACEs had a higher odds of heavy digital media use than children who had experienced less than four ACEs (aOR 1.67; 95% CI 1.43–1.95). The findings from this study are demonstrative for pediatricians that heavy digital media use may indicate a need to screen for ACEs. Programs and interventions that promote the creation of positive childhood experiences are further needed to address digital media usage concerns.

## Introduction

There has been a marked increase in the prevalence of digital media use, including tablets, smartphones, TV, and other electronic devices, among children and youth in the last twenty years, with the most marked increase occurring since 2010 (Twenge et al., 2019). Debate about the role of digital technology and how it may affect young people has led to research on the relationship between digital media use and child and adolescent development (Bell et al., 2015). Numerous studies have examined the time spent on digital media and digital communication and life satisfaction among children and adolescents, finding that children and youth with heavier digital use are also more likely to have lower life satisfaction (Topić et al., 2023; Ramírez et al., 2021). Heavy digital media use has also been associated with increased likelihood of overweight/obesity, hyperactivity, sleep problems, and lower academic performance (Haghjoo et al., 2022; Tandon et al., 2019; Twenge, Hisler et al., 2019a; Howie et al., 2020). This has led to advocacy and policy efforts around reducing digital media use in children and adolescents, with the American Academy of Pediatrics recommending that children between the ages of 5–18 years old spend no more than two hours a day using digital media (American Academy of Pediatrics, 2016).

Little is known about what factors may contribute to heavy digital media use in households. Parent, child, and household characteristics may all play a role (Lauricella et al., 2015), consistent with Bronfenbrenner’s bioecological model regarding the influence of factors existing at multiple levels of the social ecology on child development (Bronfenbrenner & Evans, 2000). For children and youth, within-family experiences may be particularly relevant for understanding heavy digital media use. The concept of traumatic experiences in children, often measured as adverse childhood experiences (ACEs), includes experiences of household (i.e. family) dysfunction, abuse, and neglect that may occur before the age of 18, have been associated with heavy digital media use (Jackson et al., 2021; Domoff et al., 2021). ACEs are often associated with parenting stress, and parenting stress may further exacerbate the use of digital media, with parents experiencing higher levels of parenting stress less likely to be able to limit digital media use (Kattein et al., 2023). For families with strong parent child interactions and attachments, these attachments may lead to increased social media monitoring through healthy amounts and content of screen media use (Holmgren et al., 2022). Compensatory internet use has been demonstrated to be a potential coping mechanism for environmental stressors among adults, which may lead to heavy digital media use in that population (Kardefelt-Winther, 2014).

For children and youth, it appears that the number of adverse childhood events experienced is an important predictor of negative future outcomes. For example, prior research has found that children with four or more ACEs had a much higher likelihood of heavy digital media use; both family resilience and connection were mediators of this relationship (Jackson et al., 2021). Exposure to ACEs has been shown to increase the likelihood of excessive screen time (Xu et al., 2024). Yet, while focus on negative childhood experiences is important when examining determinants of heavy digital media use, it may be less informative than balancing this perspective with a more salutogenic approach for informing prevention efforts.

To that end, positive childhood experiences (PCEs) are conditions that can promote positive growth and development and confer protection for balancing risk factors in predicting youth outcomes (Bethell 2019). PCEs have been defined and constructed using the Healthy Outcomes from Positive Experiences (HOPE) framework, a shift from looking at negative experiences to looking at positive experiences and resiliency, include safe, stable peer to peer and child to adult relationships, as well as having safe and supportive neighborhoods and communities to learn, play, and grow (Sege et al. 2017; Sege et al., 2024). There has not yet been an investigation of the link between PCEs and digital media use. This is needed given the relationships between adverse childhood experiences, positive childhood experiences, and child and adolescent physical and mental health (Crouch et al., 2024).

Therefore, the purpose of this study was to examine the prevalence of both adverse and positive childhood experiences and to examine how these experiences may vary by light, moderate, and heavy digital media use among children and adolescents in a nationally representative sample in the U.S. We focus on children above age six and adolescents as they are more likely developmentally to use media relatively independently (American Academy of Pediatrics, 2016). We hypothesize that school-age children and adolescents with higher rates of ACEs will be more likely to engage in heavy digital media use and that children and adolescents with PCEs will be less likely to engage in heavy digital media use. Finally, we hypothesize that children and adolescents in most need, i.e. those with high ACEs exposure and low PCEs factors, will have the highest rates of digital media use. The findings from this study will be instrumental for policy makers and program developers to design programs and interventions to decrease the use of digital media among children and adolescents who may be at high risk of heavy digital use.

## Methods

This study used cross-sectional data from the 2021–(Child 2022) National Survey of Children’s Health (NSCH), which is a mail and online survey that asks caregivers about one child in their household that is below 18 years of age. It is designed to be nationally representative of children and adolescents in the U.S. and results are discussed in terms of the child. The NSCH is sponsored by the Maternal and Child Health Bureau of the Health Resources and Service Administration. More information about the survey and the sampling process can be found at childhealthdata.org (Data Resource Center for Child and Adolescent Health, 2023). The 2021–2022 NSCH had 104,995 complete interviews. The dataset was then limited to children who were six years of age or older, as many of the positive childhood experiences questions are asked to children of school age. The final sample was 57,280 children with complete information for demographics, ACEs, PCEs, and screentime information.

The main variable of interest was digital media usage. The NSCH asks caregivers the following: “On most weekdays, about how much time does this child usually spend in front of a TV, computer, cellphone or other electronic device watching programs, playing games, accessing the internet or using social media, not including schoolwork?” Responses could include less than one hour, one hour, two hours, three hours, or four or more hours. Based on prior literature, three categories of digital media use were created: light use (less than two hours on a typical weekday), moderate use (2–3 h on a typical weekday), and heavy use (four or more hours on a typical weekday) (Kattein et al., 2023).

ACEs were defined using the 9 NSCH ACE types, which asks the caregiver if the child has ever experienced the following: economic hardship, parental divorce/separation, parental death, parental incarceration, household violence, household mental illness, household substance misuse, racial/ethnic mistreatment, and sex/gender discrimination (Crouch et al., 2019). Children with four or more ACEs are more likely to have poor mental and physical health, and engage in risky behavior, so counts of ACEs as four or more were included (Crandall et al., 2019). PCEs were also selected based on prior literature and included the following: after school activities, community volunteer, guiding mentor, connected caregiver, safe neighborhood, supportive neighborhood, and resilient family. The selection and construction of PCEs are defined in previously published research (Crouch et al., 2021).

As children and families exist within communities, we used the Bronfenbrenner bioecological model to select child, caregiver, and household characteristics that may also influence digital media use (Bronfenbrenner & Evans, 2000). These included the sex of the child (male, female), age of the child (6–12, 13–17), race/ethnicity of the child (Non-Hispanic White, Non-Hispanic Black, Hispanic, and Non-Hispanic Other), and special healthcare needs of the child, where special healthcare needs was determined using the 5-item NSCH special healthcare needs screener. Caregiver and household characteristics included primary language spoken at home (English versus not English), caregiver education (high school education or less, or college education or more), and family structure (two parents currently married, two parents not married, single parent, and other type). Federal poverty level (four categories below and above the federal poverty level) was also included.

To calculate frequencies, proportions, and unadjusted associations, descriptive statistics and bivariate analyses were utilized. Statistical significance was deemed at α = 0.05. As stated in the Methods section, all results are discussed in terms of the child, as instructed by the NSCH, as the survey is sampled and weighted to be representative of children and adolescents in the U.S. Multivariable regression models were utilized to examine the association between ACEs, PCEs, and heavy digital media use. All analyses used the sampling weights, cluster, and strata put forth by the NSCH, with additional information on childhealthdata.org. SAS statistical software was used for all analyses. This study was approved as exempt by the [name concealed for review] institutional review board.

## Results

Our sample was nearly equally distributed male and female (Table 1). Just over 57% of children were six to twelve years of age and 26.1% of the sample was Hispanic children. Nearly a quarter of the sample had at least one special healthcare need (24.8%). Less than 15% of children did not speak English as their primary language (14.8%). The majority of children had caregivers with some college education or more and resided in a family structure with two parents, currently married. Less than 18% of children lived below the federal poverty level. Nearly 30% of children had heavy digital media use, with statistically significant differences reported by sex, age, race/ethnicity, special health care needs, primary language spoken at home, education level of the caregiver, structure of the family, and poverty/income level (Table 1). The most common ACE types were divorce (27.2%), economic hardship (13.2%), household mental illness (10.0%), and household substance use (9.9%). Every other ACE type was less than 10%. Most children participated in after school activities (70.7%), resided in a resilient family (83.7%), had a guiding mentor (78.8%), lived in a safe neighborhood (67.2%), and had a connected caregiver (62.0%, Table 1).

**Table 1:** Characteristics of respondents to the 2021–2022 National survey of children’s Health, in total and stratified by digital media use, *n* = 54,256

Characteristic	All	Light Digital Media Use	Moderate Digital Media Use	Heavy Digital Media Use	*P* value	
		19.4%	50.9%	29.6%		
*Characteristics of Child*						
Sex of Child					**< 0.0001**	
Male	51.0	47.7	50.6	53.7		
Female	49.0	52.3	49.4	46.3		
Age of Child					**< 0.0001**	
6–12 years old	57.4	75.2	60.7	40.1		
13–17 years old	42.6	24.8	39.3	59.9		
Race/Ethnicity of Child					**< 0.0001**	
Non-Hispanic White	50.2	56.4	51.8	43.3		
Non-Hispanic Black	12.3	9.3	11.3	16.0		
Hispanic	26.1	21.8	26.1	29.1		
Non-Hispanic Other	11.4	12.5	10.8	11.7		
Special Health Care Needs, Yes	24.8	19.7	22.9	31.5	**< 0.0001**	
Primary Language					0.4840	
Not English	14.8	14.7	14.3	15.5		
Caregiver Education					**< 0.0001**	
Less than high school or high school	27.8	26.0	26.5	31.0		
Some college or more	72.3	74.0	73.5	69.0		
Family Structure					**< 0.0001**	
2 parents, currently married	64.7	70.5	66.2	58.4		
2 parents, not currently married	5.7	4.5	5.6	6.7		
Single parent	24.7	21.3	24.0	28.7		
Other	4.9	3.7	4.6	6.2		
Federal Poverty Level (FPL)					**< 0.0001**	
0–99% FPL	17.7	18.4	16.5	19.1		
100%−199% FPL	19.9	17.6	19.4	22.2		
200%−399% FPL	29.5	27.1	30.3	29.7		
400% FPL or above	33.0	37.0	33.8	29.0		

Bold indicates statistical significance at *p* < 0.05, calculated from Pearson’s Chi-square tests

Children who engaged in heavy digital media use were more likely to report each type of ACE than children who engaged in light digital media use (*p* < 0.0001, Table 2). On the flip side, children who engaged in heavy digital media use were less likely to report each type of PCE than children who engaged in light digital media use (*p* < 0.0001). Finally, among children with heavy digital media use, 11.0% had experienced four or more ACEs. Of those who had experienced four or more ACEs, none experienced more than three PCEs. In multivariable analysis adjusting for both child and caregiver characteristics, children who had experienced four or more ACEs had higher odds of heavy digital media use than children who had experienced less than four ACEs (aOR 1.67; 95% CI 1.43–1.95). Non-Hispanic Black children had a higher likelihood of heavy digital media use than Non-Hispanic White children (aOR 1.66; 95% CI 1.45–1.89). Children thirteen to seventeen years had a higher odds of heavy digital media use than children six to twelve years of age (aOR 2.66; 95% CI 2.46–2.88). Children with special healthcare needs had a higher likelihood of heavy digital media use than children without special healthcare needs (aOR 1.44; 95% CI 1.32–1.57). Children with a single parent had a greater odds of heavy digital media use than children with two parents currently married (aOR 1.15; 95% CI 1.04–1.28).

**Table 2:** Adverse childhood experiences (ACEs) and positive childhood experiences (PCEs) reported by respondents to the 2021–2022 National survey of children’s Health, in total and stratified by digital media use, *n* = 54,256

Characteristic	All	Light Digital Media Use	Moderate Digital Media Use	Heavy Digital Media Use	*P* value
Adverse Childhood Experiences					
Parent or guardian divorced or separated	27.2	21.2	25.3	34.3	**< 0.0001**
Parent or guardian died	3.8	3.0	3.5	4.7	**< 0.0001**
Parent or guardian served time in jail	7.5	5.6	6.4	10.6	**< 0.0001**
Saw or heard parents or adults slap, hit, kick, punch one another in the home	6.3	4.5	4.9	9.9	**< 0.0001**
Was a victim of violence or witnessed violence in neighborhood	4.6	3.4	3.4	7.4	**< 0.0001**
Lived with anyone who was mentally ill, suicidal, or severely depressed	10.0	6.4	8.6	14.8	**< 0.0001**
Lived with anyone who had a problem with alcohol or drugs	9.9	6.0	8.6	14.8	**< 0.0001**
Treated or judged unfairly because of his or her race or ethnic group	5.6	3.2	5.0	8.0	**< 0.0001**
Economic Hardship: Hard to cover basics like food or housing	13.2	8.7	11.9	18.2	**< 0.0001**
Sexual or Gender Discrimination	1.6	0.67	1.38	2.76	**< 0.0001**
Four or more ACEs	6.9	4.6	5.4	11.0	**< 0.0001**
Positive Childhood Experiences					
After school activities	70.7	76.8	73.2	62.7	**< 0.0001**
Community volunteer	29.0	38.4	33.3	27.0	**< 0.0001**
Guiding mentor	78.8	88.5	87.7	82.5	**< 0.0001**
Connected caregiver	62.0	73.4	64.1	50.8	**< 0.0001**
Safe neighborhood	67.2	73.5	68.0	61.7	**< 0.0001**
Supportive neighborhood	57.3	68.3	58.4	48.1	**< 0.0001**
Resilient family	83.7	90.5	86.0	75.2	**< 0.0001**

P values were calculated from Pearson’s Chi-square tests, *P* < 0.01 deemed statistically significant

Among children with four or more ACEs, those who participated in after school activities had lower odds of heavy digital use than those who did not participate in after school activities (aOR: 0.61; 95% CI 0.56–0.68, Table 3). Children who volunteered in their community (aOR: 0.71; 95% CI 0.62, 0.82) and who have a guiding mentor (aOR: 0.60; 95% CI 0.55, 0.66) had lower odds of heavy digital media use than those who did not. Among children with four or more ACEs, Non-Hispanic Black children had greater odds of heavy digital media use even when they participated in after school activities (aOR: 1.35; 95% CI 1.20, 1.53), volunteering in their community (aOR 1.35; 95% CI 1.19–1.52), and having a guiding mentor (aOR 1.28, 95% CI 1.15, 1.42) than Non-Hispanic White children.

**Table 3:** Prediction of heavy digital media use, by adverse childhood experiences Exposure, by respondents to the 2021–2022 National survey of children’s health *n* = 54,256

Characteristics	Predicting Heavy Digital Media Use	
	PE^a^	95% CI^b^
Less than Four ACEs	**1.67**	**1.43–1.95**
Four or More ACEs	(Referent)	(Referent)
Child Characteristics		
Race/Ethnicity		
White, Non-Hispanic	(Referent)	(Referent)
Black, Non-Hispanic	**1.66**	**1.45–1.89**
Hispanic	**1.38**	**1.22–1.55**
Other, Non-Hispanic	**1.29**	**1.17–1.44**
Child Sex		
Male	(Referent)	(Referent)
Female	0.85	0.78–0.92
Child Age		
6–11 years old	(Referent)	(Referent)
12–17 years old	**2.66**	**2.46–2.88**
Special Healthcare Needs		
Yes	**1.44**	**1.32–1.57**
No	(Referent)	(Referent)
Primary Language		
English	0.98	0.83–1.15
Not English	(Referent)	(Referent)
Characteristics of Caregiver/Household		
Guardian Education		
High school diploma or less	1.06	0.94–1.18
Some college or more	(Referent)	(Referent)
Family Structure		
Two parents, currently married	(Referent)	(Referent)
Two parents, not currently married	**1.34**	**1.11–1.61**
Single parent	**1.15**	**1.04–1.28**
Other	1.19	0.95–1.49
% Federal Poverty Level		
0–99% FPL	0.98	0.84–1.14
100%−199% FPL	1.10	0.97–1.27
200%−399% FPL	1.07	0.97–1.17
≥ 400% FPL	(Referent)	(Referent)

^a^ *PE* Point Estimate

^b^ 95% CI = 95% Wald confidence intervals; bold indicates statistical significance at *p* < 0.05

Compared to younger children, adolescents aged 13–17 years old had greater odds of heavy digital media use even when they participated in after school activities (aOR: 2.99; 95% CI 2.55, 2.99, Table 4), volunteering in their community (aOR 2.71; 95% CI 2.50–2.98), and having a guiding mentor (aOR 1.50, 95% CI 1.38, 1.64). Children with special health care needs had greater odds of heavy digital media use even when they participated in after school activities (aOR: 1.46; 95% CI 1.34, 1.60), volunteered in their community (aOR 1.50; 95% CI 1.38–1.64), and had a guiding mentor (aOR 1.47 95% CI 1.28, 1.74) than those without special healthcare needs. Compared to children with two currently married parents, children with a single parent had greater odds of heavy digital media use even when participating in after school activities (aOR: 1.31; 95% CI 1.06, 1.31), volunteering in their community (aOR 1.32; 95% CI 1.08–1.32), and having a guiding mentor (aOR 1.29, 95% CI 1.07, 1.55).

**Table 4:** Prediction of heavy media use by PCE exposure among children with 4 + ACEs

Characteristics	After School Activities		Community Volunteer		Guiding Mentor	
	PE^a^	95% CI^b^	PE^a^	95% CI^b^	PE^a^	95% CI^b^
Specific PCE Exposure						
Yes	0.61	0.56–0.68	0.71	0.62–0.82	0.60	0.55–0.66
No	(Referent)	(Referent)	(Referent)	(Referent)	(Referent)	(Referent)
Child Characteristics						
Race/Ethnicity						
White, Non-Hispanic	(Referent)	(Referent)	(Referent)	(Referent)	(Referent)	(Referent)
Black, Non-Hispanic	1.35	1.2–1.53	1.35	1.19–1.52	1.32	1.17–1.49
Hispanic	1.59	1.39–1.81	1.57	1.38–1.79	1.65	1.44–1.88
Other, Non-Hispanic	1.29	1.16–1.44	1.26	1.14–1.41	1.28	1.15–1.42
Child Sex						
Male	(Referent)	(Referent)	(Referent)	(Referent)	(Referent)	(Referent)
Female	0.86	0.80–0.93	0.85	0.79–0.92	0.87	0.80–0.94
Child Age						
6–11 years old	(Referent)	(Referent)	(Referent)	(Referent)	(Referent)	(Referent)
12–17 years old	2.99	2.55–2.99	2.71	2.50–2.98	2.94	2.71–3.19
Special Healthcare Needs						
Yes	1.46	1.34–1.60	1.50	1.38–1.64	1.47	1.28–1.74
No	(Referent)	(Referent)	(Referent)	(Referent)	(Referent)	(Referent)
Primary Language						
English	0.92	0.78–1.09	0.89	0.75–1.05	0.94	0.80–1.1
Not English	(Referent)	(Referent)	(Referent)	(Referent)	(Referent)	(Referent)
Characteristics of Caregiver/Household						
Guardian Education						
High school diploma or less	0.98	0.87–1.1	1.03	0.92–1.15	1.00	0.90–1.12
Some college or more	(Referent)	(Referent)	(Referent)	(Referent)	(Referent)	(Referent)
Family Structure						
Two parents, currently married	(Referent)	(Referent)	(Referent)	(Referent)	(Referent)	(Referent)
Two parents, not currently married	1.61	1.11–1.61	1.63	1.12–1.63	1.29	1.07–1.55
Single parent	1.31	1.06–1.31	1.32	1.08–1.32	1.16	1.05–1.28
Other	1.61	1.03–1.61	1.65	1.07–1.65	1.27	1.02–1.57
% Federal Poverty Level						
0–99% FPL	1.07	0.79–1.07	1.14	0.84–1.14	1.00	0.87–1.14
100%−199% FPL	1.19	0.92–1.19	1.25	0.97–1.25	1.12	0.99–1.26
200%−399% FPL	1.14	0.94–1.14	1.08	0.98–1.18	1.07	0.98–1.18
≥ 400% FPL	(Referent)	(Referent)	(Referent)	(Referent)	(Referent)	(Referent)

^a^ PE = Point Estimate

^b^ 95% CI = 95% Wald confidence intervals; bold indicates statistical significance at *p* < 0.05

## Discussion

This is the first study, to our knowledge, to examine the relationship of both positive and adverse childhood experiences and digital media use. While much has been discussed on the negative ramifications of excessive digital media use, less has been investigated on what factors affect heavy digital media use, including traumatic experiences, positive experiences, and child and family characteristics (Lauricella et al., 2015). Our findings are consistent with prior research that ACEs can increase the likelihood of heavy digital media use and expand upon earlier research by finding that children who have experienced PCEs are less likely to engage in heavy digital media use (Xu et al., 2024).

Importantly, heavy digital media use is common, with 30% of the school-age children and adolescents in our sample engaging in digital media use for four or more hours per day per caregiver report. After adjusting for child and family characteristics including poverty, several factors were found to be associated with this level of use. These include the number of ACEs experienced (four or more vs. fewer than four), race (Black as compared to White), presence of special health care needs, age (adolescents v children), and family structure (single v two parent family). Additionally, we found that among children who have four or more ACEs, those who have certain PCEs, including participating in after school activities, volunteering in their community, and having a guiding mentor, are less likely to have heavy digital media use. These findings suggest that PCEs can serve as important protective factors, reducing the likelihood of heavy digital media use, particularly among children who experience adversity.

Our findings are generally but not always consistent with existing literature regarding screen time among youth. Disparities in health risk factors including screen time have been found between Black and White youth, including a dampening of the protective effect of family income on youth screen time. For White youth there is a positive and inverse association between income and screen use; this inverse association is less apparent for Black youth (Assari, 2020). Youth with special health care needs may be more likely to engage in longer screen times, for example, among individuals with autism (Yuan et al., 2024). Screen time has been found to be higher among older as compared to younger youth (Twenge & Campbell, 2018). In the current study, finding an increased likelihood of heavy digital media use between children in single vs. dual parent homes is not consistent with other research. A large representative sample of youth from single parent, dual parent, and reconstituted households failed to find screen time differences (McMillan et al., 2015).

A central question for this study was the relationship between ACEs and heavy digital media use. Exposure to four or more ACEs was found to increase the likelihood of heavy digital media use. One potential explanation is that screen time may be one way for children and adolescents to cope with adversity (Kardefelt-Winther, 2014). That said, protective factors appear to be important in reducing risk of lengthy screen time and can balance risk. Youth with four or more ACEs who participated in after school or volunteer activities, or who had a mentor, were at reduced likelihood of heavy digital media use. One potential explanation is that children who grow up in what they perceive as a safe and stable environment are more likely to learn how to self-regulate and monitor their own behavior, as well as to delay gratification (Merrick et al., 2020). A second, competing explanation may be related to how out-of-school time is spent—if youth are engaged in after school or community activities, the time available for digital media use may be limited. Similarly, time spent with a mentor can reduce available time for interacting with screens. Many policy considerations and programs are needed to mitigate the effects of ACEs and to increase PCEs among children and families in order to reduce digital media use and improve overall health. Using a comprehensive framework to support positive youth outcomes and enhancing nurturing environments, Biglan and colleagues identify a number of policy solutions, including policies to reduce poverty, as well as the vast array of evidence-based interventions for families and within schools (Biglan et al., 2020). The results of the current study highlight the need for programs and policies, which promote PCEs among specific families, as the impacts of PCEs on digital media use are less pronounced among children of color, those with special health care needs, and those in single parent families. Tailored implementation of these programs could have a significant positive impact on the rates of heavy digital media use among these groups.

Since caregivers may present to clinical settings with concerns about digital media use among their children, this is an ideal setting to discuss the factors noted above (Ishtiaq et al., 2021). The American Academy of Pediatrics provides guidelines on how to discuss digital media usage and how parents may help children manage digital media usage. Pediatricians and other healthcare professionals can discuss with the caregiver the role that caregivers can take in being a mentor for media consumption, including modeling time with no devices, teaching children appropriate screen time behaviors, such as limiting screens before bed and using screens for learning (American Academy of Pediatrics, 2016). In addition, pediatricians should also encourage caregivers to have their children participate in after school activities, volunteer in their community, and have a guiding mentor, as these PCEs are known to improve the general well-being of children and lower the odds of heavy digital media use (Crouch et al., 2021).

There are several strengths to this study, including the use of a nationally representative survey sample. This is also the first study to examine relationships between ACEs, PCEs, and digital media use. Despite these strengths, limitations to this study include the cross-sectional nature of the data, so no causal inference is possible. The caregiver reporting of ACEs, PCES, and digital media use means that the data may suffer from social desirability bias, whereas the caregiver may underreport less socially desirable events. The questions in the NSCH used to measure PCEs do not exactly correlate with the BRFSS PCE questions utilized in much of the literature reporting on PCE exposure and different outcomes. The NSCH is address-based, so transient or homeless families are not included. Furthermore, the NSCH questions regarding the number of hours spent on digital media stop at “four or more,” even though recent research has demonstrated that children and adolescents may spend much more time on digital media than four hours (Rogers, 2019). This study did not examine differences across different types of digital media due to data limitations. There may be differences in type of digital media use by sociodemographic characteristics, and some forms of digital media use, such as social media, may be more closely associated with ACEs (You et al., 2021; Nagata et al., 2025). In addition, while previous research has shown that children with exposure to a larger number of ACEs have greater odds of problematic digital media use (Domoff et al., 2021; Raney et al., 2023), an additional important limitation is that the ACEs used in the current study are not exactly the same as the original 10 ACEs identified by Felitti and colleagues (Felitti et al., 1998). Thus, caution is warranted when comparing findings of this study with prior research on ACEs and impact on heavy digital medial use. In particular, the inclusion of economic hardship, racial/ethnic mistreatment, and sex/gender discrimination as adverse childhood experiences, while used in prior research (Crouch et al., 2019), may have impacted results obtained. An important final limitation is that the chronology of exposure to ACEs and PCEs is not known, thus rendering mediation or moderation analyses inappropriate.

## Conclusions

The findings from this study can be used in the design of interventions for children and families that encourage appropriate digital media usage. These findings are demonstrative for policymakers and program developers that programs and interventions that promote the creation of PCEs are further needed to address digital media usage concerns. Additional studies are also needed to determine the less protective effect of PCEs on digital media use among specific populations.

Future research is needed to better understand how exposure to particular combinations of both adverse and more protective positive childhood experiences relates to digital media use, and a more nuanced evaluation of type of digital media use is needed. Not all digital media use is negative; and not all adverse and positive childhood experiences are equivalent.

## Data Availability

No datasets were generated or analysed during the current study.
